# Orphan receptor GPR15/BOB is up-regulated in rheumatoid arthritis

**DOI:** 10.1016/j.cyto.2014.02.015

**Published:** 2014-06

**Authors:** Alison Cartwright, Caroline Schmutz, Ayman Askari, Jan-Herman Kuiper, Jim Middleton

**Affiliations:** a Institute of Science and Technology in Medicine, Keele University at Leopold Muller Arthritis Research Centre, RJAH Orthopaedic Hospital, Oswestry, Shropshire, United Kingdom; b Division of Immunity and Infection, University of Birmingham, Birmingham, United Kingdom; c Faculty of Medicine and Dentistry, School of Oral and Dental Sciences, Bristol University, Bristol, United Kingdom

**Keywords:** Orphan receptor GPR15/BOB, Rheumatoid arthritis, Monocyte/macrophage, Neutrophils, Inflammation

## Abstract

Chemokine receptors on leukocytes mediate the recruitment and accumulation of these cells within affected joints in chronic inflammatory diseases such as rheumatoid arthritis (RA). Identification of involved receptors offers potential for development of therapeutic interventions. The objective of this study was to investigate the expression of orphan receptor GPR15/BOB in the synovium of RA and non-RA patients and in peripheral blood of RA patients and healthy donors.

GPR15/BOB protein and messenger RNA expression were examined in RA and non-RA synovium by immunofluorescence and reverse-transcription polymerase chain reaction (RT-PCR) respectively. GPR15/BOB expression on peripheral blood leukocytes was analysed by flow cytometry and GPR15/BOB messenger RNA was examined in peripheral blood monocytes by RT-PCR.

GPR15/BOB protein was observed in CD68+ and CD14+ macrophages in synovia, with greater expression in RA synovia. GPR15/BOB protein was expressed in all patient synovia whereas in non-RA synovia expression was low or absent. Similarly GPR15/BOB messenger RNA was detected in all RA and a minority of non-RA synovia. GPR15/BOB protein was expressed on peripheral blood leukocytes from RA and healthy individuals with increased expression by monocytes and neutrophils in RA. GPR15/BOB messenger RNA expression was confirmed in peripheral blood monocytes.

In conclusion GPR15/BOB is expressed by macrophages in synovial tissue and on monocytes and neutrophils in peripheral blood, and expression is up-regulated in RA patients compared to non-RA controls. This orphan receptor on monocytes/macrophages and neutrophils may play a role in RA pathophysiology.

## Highlights

• Expression of orphan receptor GPR15/BOB was examined in RA and non-RA subjects.
• GPR15/BOB protein was observed on macrophages in synovia and increased in RA.
• GPR15/BOB messenger RNA was detected in all RA and a minority of non-RA synovia.
• GPR15/BOB protein increased on blood monocytes and neutrophils in RA.
• The orphan receptor is up-regulated in a chronic inflammatory disease.

## 1 Introduction

In rheumatoid arthritis (RA) the synovial membrane undergoes infiltration by monocytes/macrophages, T cells and B cells, and neutrophils accumulate in the synovial fluid, and these cells are centrally involved in disease mechanisms [1–4]. GPR15/BOB is an orphan chemokine receptor whose natural ligand is unknown. It was found by expression cloning of simian immunodeficiency virus (SIV) receptors which isolated two genes encoding G protein-coupled receptors (GPCRs). These proteins were named Bonzo (STRL33/CXCR6) and BOB (brother of Bonzo) and presented similarities to chemokine receptors [5]. BOB is 360 amino acids in size and is identical to the previously cloned orphan receptor, GPR15 [6]. GPR15/BOB exhibits the primary structure of a 7-transmembrane domain protein and shares sequence identity with regions of the angiotensin II receptor and GPCRs CXCR2, CXCR4, CCR5, DEZ (chemerin receptor), GPR1 and APJ (apelin receptor) [6,7]. GPR15/BOB and CCR5 share a sequence of three tyrosine residues in the amino-terminal region, which if altered in CCR5, results in decreased efficiency of infection by SIV and macrophage-tropic human immunodeficiency virus (HIV)-1 strains [7].

GPR15/BOB is a co-receptor for simian immunodeficiency virus (SIV) and human immunodeficiency virus types 1 and 2 (HIV-1 and HIV-2) [7,8]. The majority of HIV-2 envelope glycoproteins and a minority of HIV-1 envelope glycoproteins are able to use GPR15/BOB as a co-receptor, but inefficiently when compared to CCR5 or CXCR4 [5,9,10]. SIV uses GPR15/BOB to infect cell lines transfected to express the receptor. GPR15/BOB is expressed on a number of human B and T cell lines including the T/B cell hybrid cell line CEMx174. GPR15/BOB is required for SIV infection of CEMx174 [5,9,11]. It is therefore probable that GPR15/BOB does not function as an efficient co-receptor for HIV-1 *in vivo* and is more likely to serve as a co-receptor for SIV. GPR15/BOB is further suggested to be involved in HIV enteropathy, a condition whereby patients with HIV infection develop malabsorption and increased intestinal permeability with diarrhoea [12,13].

GPR15/BOB is highly expressed on the surface of neutrophils from Chinese rhesus macaques and its ligation during early SIV infection induces neutrophil death and is associated with neutropenia and disease progression [14]. Increased neutrophil apoptosis and neutropenia have also been reported in HIV patients [14].

GPR15/BOB mRNA was detected in spleen, at high levels in colon and low levels in small intestine and thymus [5]. GPR15/BOB protein has been detected on subsets of CD4+ and CD8+ T cells and also CD19+ B cells from healthy human peripheral blood mononuclear cell preparations [11]; it is also expressed by human alveolar macrophages [7]. GPR15/BOB protein has been demonstrated in small intestinal and colonic mucosa, prostate, testis and liver. In the small intestine it was expressed by lymphocytes and abundant expression was observed on the basolateral surfaces of the epithelium [12,15]. Recently GPR15/BOB was shown to regulate the homing of T cells, particularly FOXP3(+) regulatory T cells (Tregs), to the large intestine lamina propria. This indicates the role for the receptor in leukocyte recruitment in inflammation [16]. However, GPR15/BOB expression in chronic inflammatory disease has not been studied.

In a microarray study we found evidence for expression of GPR15/BOB mRNA in human RA synovium [17]. In the present study we have substantiated and continued this initial work and examined the mRNA expression and cellular source of GPR15/BOB in synovium by RT-PCR and immunofluorescence microscopy. We have further examined the expression of GPR15/BOB in RA peripheral blood. This study shows expression of GPR15/BOB in a chronic inflammatory autoimmune disease with up-regulation in RA pathology.

## 2 Material and methods

### 2.1 Ethics

Ethical approval for the study was obtained from Shropshire and Staffordshire Local Research Ethics Committee (reference 03/72/RJH and 03/73/RJH) and patients provided written informed consent.

### 2.2 Synovial tissue

Synovial tissue samples were obtained from RA patients undergoing knee replacement surgery at the Robert Jones & Agnes Hunt Orthopaedic Hospital in Oswestry. RA patients (*n* = 13; 2 male and 11 female; average age 66.8 years, range 33–79 years; disease duration ranging from 3 to 39 years) were taking disease-modifying drugs (methotrexate, etanercept, hydroxychloroquine or leflunomide) or steroids, had raised erythrocyte sedimentation rate (ESR) and C-reactive protein (CRP) and fulfilled the American College of Rheumatology criteria for classification of the disease [18]. Control synovia were from non-RA patients (*n* = 9; 6 male and 3 female; average age 54.8 years, range 41–58 years) who had no history of RA, were medication free and undergoing knee arthroscopy with symptoms of suspected articular cartilage, meniscal or anterior cruciate ligament damage or degeneration.

RA synovia demonstrated classic RA histopathology with thickening of the lining layer and mononuclear infiltration of the sub-lining including formation of perivascular infiltrates. Non-RA synovia were obtained from arthroscopically non-inflamed sites and were histologically normal in appearance.

### 2.3 Peripheral blood

Peripheral blood (PB) was obtained from RA patients with active disease as defined by raised ESR and CRP, *n* = 11 (2 male and 9 female; average age 61.9 years, range 38–94 years; disease duration ranging from 6 months to 24 years) who were taking non-steroidal anti-inflammatory drugs, disease modifying drugs or steroids. 4 patients were rheumatoid factor (RF) positive, 4 were RF negative and 3 of unknown RF status. Control samples were obtained from healthy donors with no inflammatory conditions, *n* = 11 (4 male and 7 female; average age 51.8 years, range 28–78 years); 7 were RF negative and 4 of unknown RF status.

### 2.4 Leukocyte isolation

Leukocytes were isolated from EDTA-treated PB by lysing the erythrocytes for 15 min using an ice-cold isotonic solution of ammonium chloride. The leukocytes were then collected by centrifugation.

### 2.5 Monocyte/macrophage isolation

Healthy PB was collected into preservative-free heparin and mononuclear cells were isolated by gradient centrifugation with Ficoll-Paque Plus (Amersham-Biosciences, Little Chalfont, UK). Monocytes/macrophages were further isolated by the standard procedure of adherence onto a glass dish which was carried out for 30–45 min at 37 °C, 5% CO_2_ [19]. Non-adherent cells were washed off leaving monocytes/macrophages which were then lysed for RT-PCR.

### 2.6 Flow cytometry

Isolated leukocytes were analysed for cell surface expression of chemokine receptors and cluster of differentiation (CD) markers by flow cytometry. Non-specific binding by Fc receptors was blocked with 10% high purity human immunoglobulin (Flebogamma; Grifols, Cambridge, UK Ltd.). Cells (3 × 10^5^) were then stained for 30 min with antibody against chemokine receptors, CD markers or isotype controls, with each primary antibody tested in a separate tube. The antibodies used were anti-human GPR15/BOB mouse monoclonal antibody (10 μg/ml, MAB3654; clone 367902; R&D Systems, Abingdon, UK), anti-human CD14 mouse monoclonal antibody (5 μg/ml, 555396; BD Biosciences, Oxford, UK), anti-human CD3 mouse monoclonal antibody (15 μg/ml, M0835; Dako, Ely, UK) and anti-human CXCR1 mouse monoclonal (25 μg/ml, MAB330; R&D Systems) and isotype controls IgG1, IgG2a and IgG2b (all from Dako), all in 2% bovine serum albumin (BSA)/phosphate buffered saline (PBS). After washing with 2%BSA/PBS, cells were further incubated for 30 min with either goat anti-mouse IgG2b-RPE (for GPR15/BOB), goat anti-mouse IgG2a-RPE (for CD14 and CXCR1), or goat anti-mouse IgG-FITC (for CD3) secondary antibodies (all Cambridge Biosciences, Cambridge, UK). Following washing samples were analysed on a FACScan flow cytometer with CellQuestPro software (Becton Dickinson, Oxford, UK). Leukocyte populations were identified by their forward scatter (FSC) versus side scatter (SSC) profiles and by their strong expression of CD14 (monocytes), CD3 (T cells) or CXCR1 (neutrophils).

### 2.7 Immunofluorescence labelling of synovial tissue sections

Immunofluorescence staining of synovial tissue cryosections (6 μm thick) was performed with sections fixed in acetone for 10 min on ice, rinsed in PBS and incubated for 1 h with anti-human GPR15/BOB antibody (5 μg/ml; as above). For double label experiments anti-GPR15/BOB antibody was incubated together with mouse monoclonal antibodies to either CD14 (1 μg/ml; clone TÜK4), CD68 (9 μg/ml; clone EBM11), CD20 (2 μg/ml; clone L26), CD3 (6 μg/ml; clone F7.2.38) (all from Dako) or CD138 (2 μg/ml; clone B-A38; Serotec) all in PBS. Detection of anti-GPR15/BOB antibody was with Alexa 488 goat anti-mouse IgG2b. Antibodies to CD14 and CD20 were detected with Alexa 594 goat anti-mouse IgG2a and antibodies to CD68, CD3 and CD138 were detected with Alexa 594 goat anti-mouse IgG1 secondary antibody. All secondary antibodies (Molecular Probes, Invitrogen, Paisley, UK) were diluted 1:400 in PBS containing 10% human serum. Nuclear staining was performed with 4′,6-diamidino-2-phenylindole dihydrochloride (DAPI; 2 μg/ml in PBS; Sigma, Poole, UK) for 3 min before mounting. Controls were performed using isotype-matched IgGs (Dako) in place of primary antibodies, followed by respective Alexa secondary antibodies.

### 2.8 Reverse transcription-polymerase chain reaction (RT-PCR)

Total RNA was extracted from frozen blocks of synovia using TRIreagent solution (Sigma, Poole, UK) or from isolated monocytes/macrophages using RNAqueous kit (Ambion, Applied Biosystems, Warrington, UK) according to the manufacturer’s instructions. The quantity recovered was determined by spectrophotometry and the integrity was assessed by agarose gel electrophoresis. Total RNA (1 μg) was reverse transcribed using oligo(dT) primers (MWG Biotech, Ebersberg, Germany) and MMLV reverse transcriptase (Promega, Southampton, UK) at 37 °C for 1 h. The reactions were then heated to 70 °C for 5 min to inactivate the enzyme, placed on ice and 60 μl H_2_O was added. Appropriate dilutions of the resulting cDNA were then used for semi-quantitative PCR using specific primers for GPR15/BOB [F 5′-GTG ATG GAC CCA GAA GAA AC-3′; R 5′-GGA CAG AAG AGT AGG CAA CC-3′ (515 base pairs (bp); GenBank:NM005290); MWG Biotech]. PCR primers were run through a BLAST program to ensure gene specificity. The PCR reactions were normalised against the ribosomal RNA L27 using specific primers [F 5′-GAC GCA AAG CTG TCA TCG TG-3′; R 5′-GCA GTT TCT GGA AGA ACC AC-3′ (344 bp; GenBank:BC007273); MWG Biotech]. The annealing temperature was 57 °C for each primer pair.

### 2.9 Statistical analysis

GraphPad Prism Version 5.01 was used for all statistical analysis. Expression of GPR15/BOB protein was analysed on synovial cryosections by unpaired t test. Levels of receptor protein (mean fluorescence intensities) on PB leukocytes were analysed by Mann Whitney test and percentage positive PB leukocytes were analysed by unpaired t test.

## 3 Results

### 3.1 GPR15/BOB expression in synovium

**Fig. 1:**
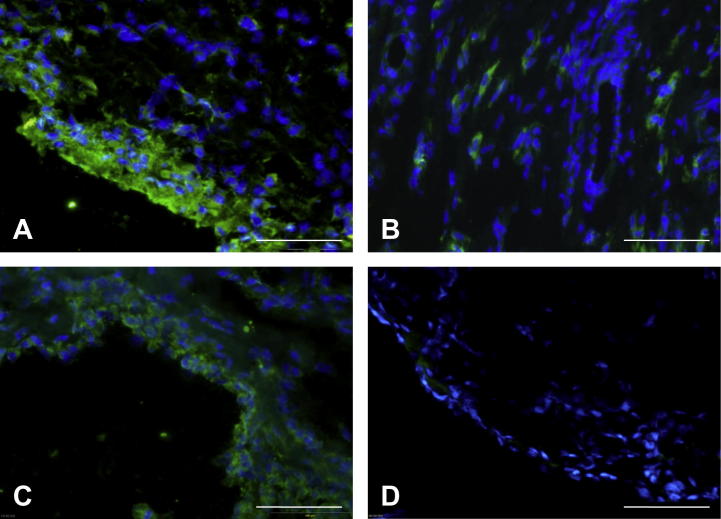
Expression of GPR15/BOB in RA and non-RA synovial tissue. Cryosections were incubated with antibody to GPR15/BOB and DAPI nuclear stain. The photomicrographs show staining for GPR15/BOB (green) and DAPI (blue) in (A) RA synovial lining, (B) RA sub-lining, (C) non-RA lining; and (D) RA isotype control. The bar represents 100 μm.

Expression of GPR15/BOB was examined in synovial tissue cryosections by immunofluorescence staining. The receptor was detected in all RA patients examined (*n* = 13) in both lining (Fig. 1A) and sub-lining layers (Fig. 1B) where expression was generally strong, localising to the cell membrane and cytoplasm. The proportion of stained cells varied between patients from a few scattered positive cells to widespread positivity. By contrast, in non-RA synovial sections (*n* = 9 individuals) weaker expression of GPR15/BOB was observed in the lining layer (Fig. 1C) and was essentially negative in the sub-lining. No staining was observed using the isotype controls for all antibodies used (Fig. 1D).

**Fig. 2:**
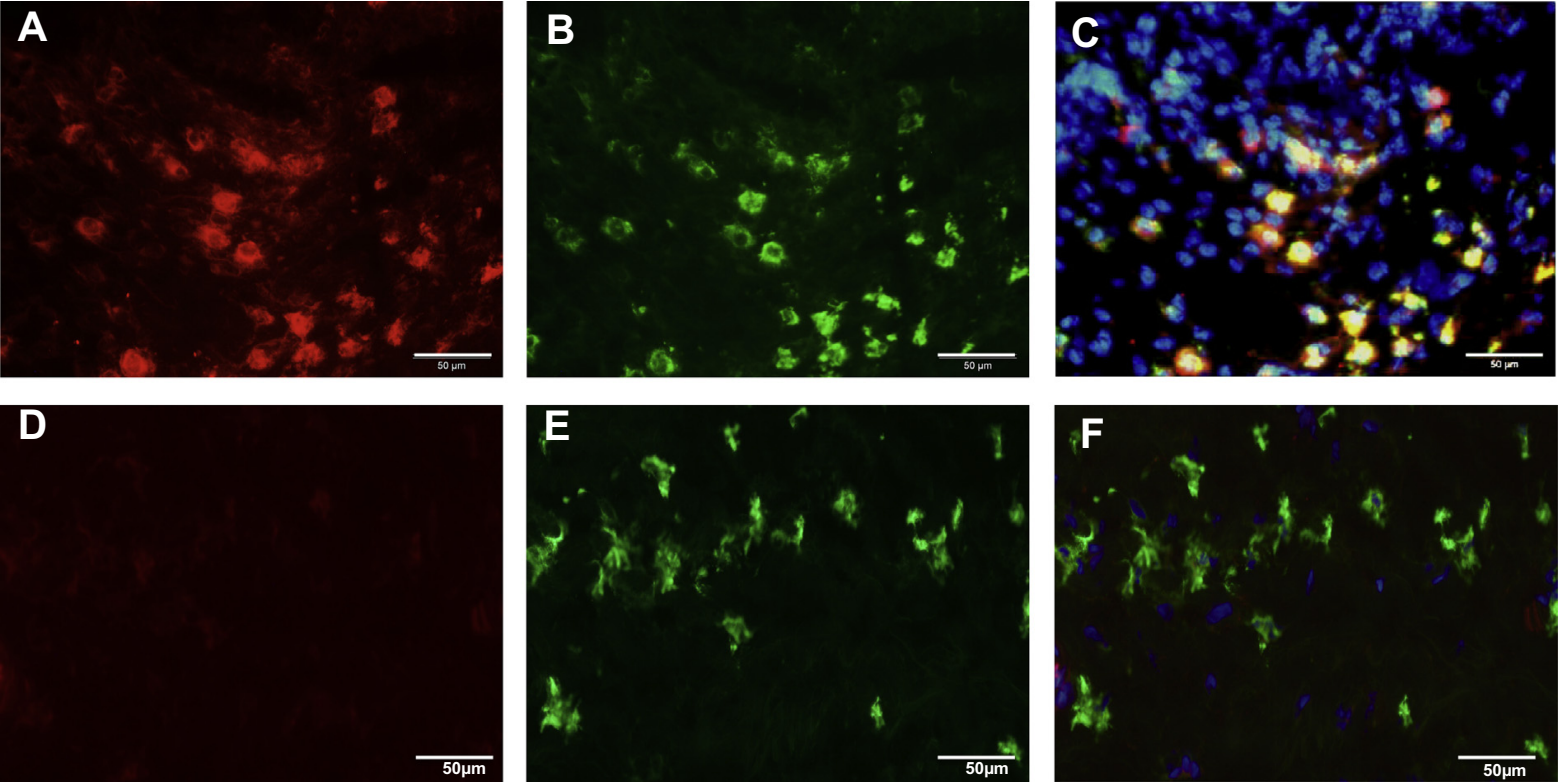
Expression of GPR15/BOB and CD68 in RA and non-RA synovium. Cryosections were stained with antibodies to GPR15/BOB and macrophage marker CD68. (A) Rheumatoid synovial sub-lining stained with anti-GPR15/BOB (red), and (B) stained with anti-CD68 (green). (C) Merge of images (A) and (B) with DAPI displaying cells with co-localised GPR15/BOB and CD68 (yellow). (D) Non-RA sub-lining stained with anti-GPR15/BOB (red) showing lack of expression, and (E) stained with anti-CD68 (green). (F) Merge of images (D) and (E) with DAPI. The bar represents 50 μm.

To identify the cell type expressing GPR15/BOB, double label immunofluorescence was carried out using antibodies to GPR15/BOB and markers for monocytes/macrophages, T cells, B cells and plasma cells; neutrophils are rarely present in synovial tissue and were not examined [20]. GPR15/BOB was present on monocytes/macrophages identified by the specific marker CD68 (Fig. 2A–C) in all RA synovia examined (CD68, *n* = 11). GPR15/BOB co-localised with CD68 expression in the sub-lining and lining layers. Non-RA synovia (*n* = 5) exhibited co-localisation of GPR15/BOB with CD68 positive cells in the lining but not in the sub-lining which was basically GPR15/BOB negative (Fig. 2D–F). CD14+ cells also colocalised with GPR15/BOB and agreed with the CD68 results, suggesting macrophages were positive for the receptor (data not shown). No co-localisation was observed between GPR15/BOB and CD3 (T lymphocytes) or CD20 (B lymphocytes) in either RA or non-RA synovia (data not shown). Non-RA synovium demonstrated limited staining with CD3 and occasional staining with CD20. Co-localisation was effectively negative between GPR15/BOB and CD138 (plasma cells) in RA synovia. Double labelling of GPR15/BOB and CD138 was not carried out on non-RA tissue as plasma cells are not present in non-infiltrated synovium. Tissue sections stained with isotype controls in place of primary antibodies were negative in each case (data not shown).

**Fig. 3:**
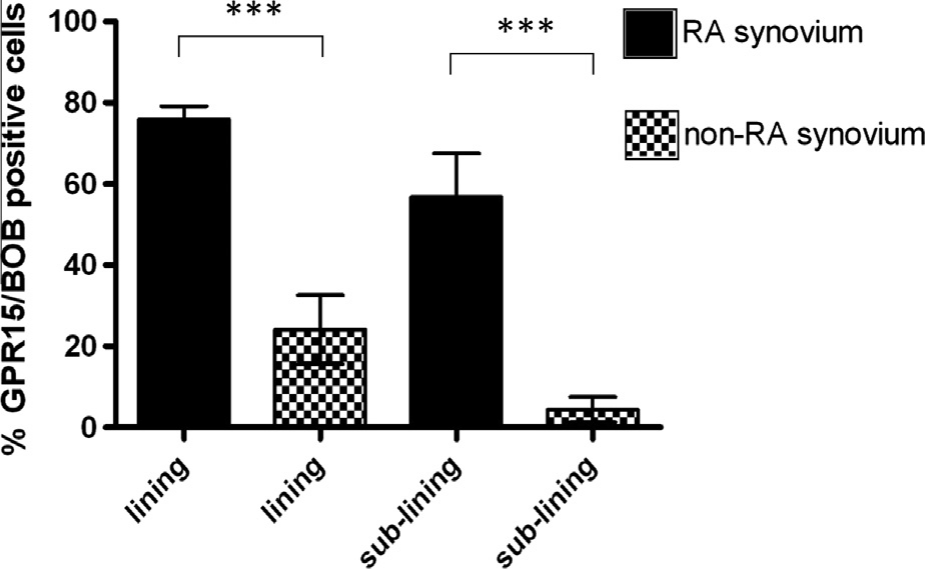
Percentage GPR15/BOB positive cells in synovial lining and sub-lining from RA and non-RA patients. The percentage (mean ± standard deviation) of total cells (DAPI+) that expressed GPR15/BOB in the lining and sub-lining layers of RA (*n* = 4) and non-RA tissue (*n* = 3) was significantly higher in RA synovium in comparison to non-RA in both the lining (*p* = 0.0003) and sub-lining layers (*p* = 0.0005) by unpaired t test. Cells were counted in 5 randomly selected fields of view using magnification of 600×. *** = *P* < 0.001.

To quantitate the difference in expression of GPR15/BOB in RA and non-RA synovia the number of GPR15/BOB+ and DAPI+ cells were counted in the lining and sub-lining layers of both RA and non-RA tissue (Fig. 3). The percentage of total cells (DAPI+) that expressed GPR15/BOB was significantly higher in RA synovium in comparison to non-RA in both the lining and sub-lining layers.

### 3.2 GPR15/BOB expression by PB monocytes and neutrophils

**Fig. 4:**
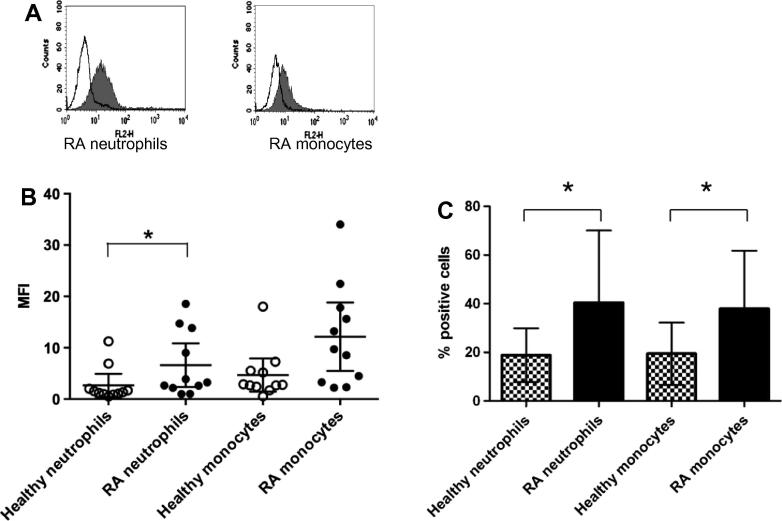
GPR15/BOB expression by neutrophils and monocytes in RA peripheral blood (PB) using flow cytometry. (A) Representative histograms showing GPR15/BOB expression by neutrophil and monocyte populations (solid fill), empty fill histograms represent the isotype-matched control. (B) GPR15/BOB expression as measured by mean fluorescence intensity (MFI; mean with 95% confidence interval) increased significantly on RA PB neutrophils (*p* = 0.030) and with an increase close to significance on RA PB monocytes (*p* = 0.056) when compared to these cell populations from healthy donors. Data were analysed by Mann Whitney test. (C) RA neutrophil and monocyte populations also exhibited a significant increase in percentage of cells expressing GPR15/BOB (*p* = 0.035 and *p* = 0.034 respectively) when compared to these cell populations from healthy donors. The data represent percentage means ± standard deviation and were analysed by unpaired t tests. Data were from healthy donors (*n* = 11) and RA patients (*n* = 11). *=*P* < 0.05.

GPR15/BOB expression by leukocytes from RA and healthy PB was examined by flow cytometry. Leukocyte populations were identified by their FSC/SSC profiles and by their strong cell surface expression of characteristic markers: CD14 on monocytes, CD3 on T lymphocytes and CXCR1 on neutrophils. GPR15/BOB was detected on monocytes and neutrophils (Fig. 4A). A significant increase in GPR15/BOB expression as measured by mean fluorescence intensity (MFI) (Fig. 4B) was observed on RA PB neutrophils and an increase close to significance was observed on RA PB monocytes when compared to these cell populations from healthy donors. Furthermore a significant increase in the percentage of cells expressing GPR15/BOB was observed in RA neutrophil populations and also in RA monocyte populations (Fig. 4C). GPR15/BOB was also detected on the surface of lymphocytes but expression was comparable between RA and healthy donors (data not shown).

### 3.3 RT-PCR analysis of GPR15/BOB

**Fig. 5:**
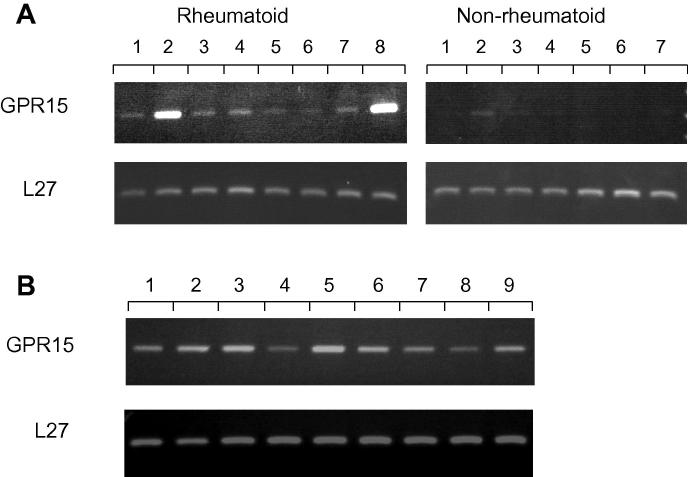
GPR15/BOB mRNA in synovial tissue and monocytes/macrophages by RT-PCR. (A) RNA isolated from synovial tissue of RA patients (*n* = 8) and non-RA controls (*n* = 7) showing the presence of GPR15/BOB mRNA. (B) GPR15/BOB mRNA expression by monocytes/macrophages isolated from PB of healthy donors (*n* = 9). PCR reactions were normalised using L27 ribosomal protein.

Expression of GPR15/BOB mRNA was analysed in RA (*n* = 8) and non-RA (*n* = 7) synovia by RT-PCR to confirm observations from immunofluorescence staining. GPR15/BOB mRNA was detected in all RA patients examined although the band intensity varied between individuals (Fig. 5A), being considerably stronger in patients 2 and 8. GPR15/BOB mRNA was barely detectable in non-RA synovia, being detected in only one (patient 2) out of seven non-RA patients examined. L27 ribosomal gene was used to normalise PCR reactions to allow comparison between samples.

To confirm monocytes/macrophage expression of GPR15/BOB, RT-PCR was performed on these cells isolated from the PB of healthy donors (*n* = 9). GPR15/BOB mRNA was detected in isolated monocytes/macrophages from these individuals (Fig. 5B).

## 4 Discussion

Infiltration of the synovial membrane by inflammatory leukocytes is a characteristic pathological feature in rheumatoid arthritis patients. Leukocytes including macrophages are recruited by the action of chemoattractant cytokines secreted within the synovium by both resident and infiltrated cells [4]. In this study we demonstrated that the GPR15/BOB receptor was expressed by macrophages in synovial tissue with up-regulation in RA tissue. In circulating blood both monocytes and neutrophils expressed GPR15/BOB. Percentages of GPR15/BOB+ cells were significantly raised in RA blood and the abundance of GPR15/BOB on these cells was also greater in RA in comparison to healthy donor blood. This suggests that in RA there may be preferential recruitment of GPR15/BOB+ monocytes to the synovium or alternatively GPR15/BOB may be up-regulated on monocytes, via the actions of pro-inflammatory cytokines, once the monocytes have reached the synovium.

Examination of synovial tissue mRNA expression suggested GPR15/BOB was expressed in all RA patients examined. The mRNA band intensity appeared greatest in patients 2 and 8 (Fig. 5A) which may be related to their elevated disease severity and absence of DMARD or steroid therapies. However, real-time PCR would be needed to confirm differences in GPR15/BOB expression between RA patients.

GPR15/BOB mRNA was barely or not detected in non-RA synovium. In addition, GPR15/BOB protein was detected at low level in the synovial lining layer and was essentially negative in the sub-lining. We have therefore shown that GPR15/BOB is up-regulated in RA synovia compared to non-RA controls. A previous initial study by our group using gene microarrays of synovial tissue showed that GPR15/BOB mRNA expression was present in RA and not detected in non-RA [17] and the results of the present study are therefore in agreement with and extend this earlier report.

Chemokine receptor expression is regulated as a result of signal transduction cascades which occur following the ligation of cell surface receptors by cytokines such as TNFα which are known to increase in the circulation in RA [21]. The subsequent transcriptional activation of genes involved in inflammation leads to production of pro-inflammatory cytokines, chemokines and increased expression of cell surface receptors. Therefore elevated cytokines in RA may be up-regulating GPR15/BOB on monocytes/macrophages and neutrophils. In this connection, expression of CCR9 was found to increase significantly on THP-1 monocytic cells following TNFα stimulation [22].

Cell surface expression of GPR15/BOB may be induced by extracellular signals that activate the PI3-kinase pathway and increase binding of 14-3-3 (a scaffold protein involved in signaling complex formation) to GPR15/BOB leading to its subsequent expression on the cell-surface [23]. The increase in expression of GPR15/BOB on monocytes in PB may be related to the increased expression of GPR15/BOB in RA synovium in that PB monocytes bearing the receptor may be recruited from the blood into the synovium in RA, possibly involving an unknown ligand to GPR15/BOB.

GPR15/BOB is expressed by CD4+ T cells [7] and although PB lymphocytes expressed GPR15/BOB in this study they were not positive in the synovium suggesting that GPR15/BOB+, CD4+ T cells were not recruited to the synovium via a GPR15/BOB ligand. However, neutrophils bearing GPR15/BOB may be recruited into the RA joint. Neutrophils are not present in the RA synovium to any appreciable extent [24], but they are present in synovial fluid and have been found to express GPR15/BOB in RA patients [unpublished observation by Dr Caroline Schmutz]. Therefore increased expression of GPR15/BOB on PB neutrophils in RA may be involved in disease pathology.

Interestingly, SIV envelope-ligation of GPR15/BOB on the surface of circulating neutrophils from Chinese rhesus macaques was found to induce neutrophil apoptosis during SIV infection [14]. HIV patients also suffer from increased neutrophil apoptosis in chronic disease and the rate of neutrophil apoptosis and associated neutropenia is related to rate of disease progression [14]. A role for GPR15/BOB in regulating neutrophil apoptosis in RA is currently unknown.

Macrophages play a major role in the pathogenesis of RA due to their production of pro-inflammatory cytokines including TNFα and IL-1β, inflammatory chemokines including CXCL8 and CCL2, and also degradative enzymes. These factors all contribute to synovial inflammation and joint erosion. Neutrophils accumulate in the synovial fluid in RA where they become activated, releasing proteases and lysozomal enzymes leading to cartilage damage, and also pro-inflammatory cytokines and chemokines including IL-1, CXCL8 and CCL3 [25,26]. The GPR15/BOB receptor on monocytes/macrophages and neutrophils may therefore play a role in RA by attracting these cells into the synovial joint in response to unknown ligand(s) to GPR15/BOB. Since macrophages are important cells in RA pathology GPR15/BOB may provide an interesting therapeutic target in the treatment of inflammation and joint destruction in patients, and further work on the function of this receptor in RA would be of interest. A recent study by Kim et al. [16] has found the presence of GPR15/BOB on T cells, especially FOXP3(+) regulatory T cells, which regulated homing of these cells to the large intestine, leading to altered inflammation. In the current study GPR15/BOB was mainly expressed by monocytes/macrophages in the RA synovium rather than T cells. Therefore there may be tissue-specific differences in the expression and role of GPR15/BOB.

## Funding

This work was supported by Keele University and the Wellcome Trust.

## References

[1] Ma Y., Pope R.M. (2005). The role of macrophages in rheumatoid arthritis. Curr Pharm Des.

[2] Kinne R.W., Stuhlmüller B., Burmester G.R. (2007). Cells of the synovium in rheumatoid arthritis: macrophages. Arthritis Res Ther.

[3] Mulherin D., Fitzgerald O., Bresnihan B. (1996). Synovial tissue macrophage populations and articular damage in rheumatoid arthritis. Arthritis & Rheum.

[4] Szekanecz Z., Vegvari A., Szabo Z., Koch A.E. (2010). Chemokines and chemokine receptors in arthritis. Front Biosci (Schol Ed).

[5] Deng H.K., Unutmaz D., KewalRamani V.N., Littman D.R. (1997). Expression cloning of new receptors used by simian and human immunodeficiency viruses. Nature.

[6] Heiber M., Marchese A., Nguyen T., George S.R., O’Dowd B.F. (1996). A novel human gene encoding a G-protein-coupled receptor (GPR15) is located on chromosome 3. Genomics.

[7] Farzan M., Choe H., Martin K. (1997). Two orphan seven-transmembrane segment receptors which are expressed in CD4-positive cells support simian immunodeficiency virus infection. J Exp Med.

[8] Elliott S., Riddick N., Francella N. (2012). Cloning and analysis of sooty mangabey alternative coreceptors that support simian immunodeficiency virus SIVsmm entry independently of CCR5. J Virol.

[9] Edinger A.L., Hoffman T.L., Sharron M., Lee B., O’Dowd B., Doms R.W. (1998). Use of GPR1, GPR15, and STRL33 as coreceptors by diverse human immunodeficiency virus type 1 and simian immunodeficiency virus envelope proteins. Virology.

[10] Pohlmann S., Stolte N., Munch J. (1990). Co-receptor usage of BOB/GPR15 in addition to CCR5 has no significant effect on replication of simian immunodeficiency virus in vivo. J Infect Dis.

[11] Kiene M., Marzi A., Urbanczyk A. (2012). The role of the alternative coreceptor GPR15 in SIV tropism for human cells. Virology.

[12] Clayton F., Kotler D.P., Kuwada S.K. (2001). Gp120-induced Bob/GPR15 activation: a possible cause of human immunodeficiency virus enteropathy. Am J Pathol.

[13] Maresca M., Mahfoud R., Garmy N., Kotler D.P., Fantini J., Clayton F. (2003). The virotoxin model of HIV-1 enteropathy: involvement of GPR15/Bob and galactosylceramide in the cytopathic effects induced by HIV-1 gp120 in the HT-29-D4 intestinal cell line. J Biomed Sci.

[14] Elbim C., Monceaux V., Mueller Y.M. (2008). Early divergence in neutrophil apoptosis between pathogenic and nonpathogenic simian immunodeficiency virus infections of nonhuman primates. J Immunol.

[15] Li Q., Estes J.D., Duan L. (2008). Simian immunodeficiency virus-induced intestinal cell apoptosis is the underlying mechanism of the regenerative enteropathy of early infection. J Infect Dis.

[16] Kim S.V., Xiang W.V., Kwak C. (2013). GPR15-mediated homing controls immune homeostasis in the large intestine mucosa. Science.

[17] Schmutz C., Hulme A., Burman A. (2005). Chemokine receptors in the rheumatoid synovium: upregulation of CXCR5. Arthritis Res Ther.

[18] Arnett F.C., Edworthy S.M., Bloch D.A. (1998). The American Rheumatism Association 1987 revised criteria for the classification of rheumatoid arthritis. Arthritis Rheum.

[19] Wahl L., Wahl S., Smythies L., Smith P.D. (2005). Isolation of human monocyte populations. Curr Protocols Immunol Suppl.

[20] Firestein GS. Rheumatoid synovitis and pannus. In: Klippel JH, Dieppe PA, editors. Rheumatology, vol. 1, 2nd ed. Mosby; 1998. p. 13.1–13.24.

[21] Szekanecz Z., Koch A.E., Kunkel S.L., Strieter R.M. (1998). Cytokines in rheumatoid arthritis. Potential targets for pharmacological intervention. Drugs Aging.

[22] Schmutz C., Cartwright A., Williams H. (2010). Monocytes/macrophages express CCR9 in rheumatoid arthritis and CCL25 stimulates their differentiation. Arthritis Res Ther.

[23] Chung J.J., Okamoto Y., Coblitz B., Li M., Qiu Y., Shikano S. (2009). PI3/Akt signalling-mediated protein surface expression sensed by 14-3-3 interacting motif. FEBS J.

[24] Tak P.P., Firestein G.S., Panayi G.S., Wollheim F.A. (2000). Examination of the synovium and synovial fluid. Rheumatoid arthritis: frontiers in pathogenisis and treatment.

[25] Scapini P., Lapinet-Vera J.A., Gasperini S. (2000). The neutrophil as a cellular source of chemokines. Immunol Rev.

[26] Cross A., Barnes T., Bucknall R., Edwards S.W., Moots R.J. (2006). Neutrophil apoptosis in rheumatoid arthritis is regulated by local oxygen tensions within joints. J Leukocyte Biol.

